# Health Versus Disease

**Published:** 1920-06-12

**Authors:** 


### THE MATRONS' AND SISTERS' DEPARTMENT.

### HEALTH VERSUS DISEASE.

No part of the Interim Report on the Future Provision of Medical and Allied Services is more stimulating than that relating to Physical Culture. Florence Nightingale's ideals for the nursing service foreshadowed the nurse as a radiating centre of health. While never losing sight of the nurse as combatant against disease, she seems to have regarded this aspect of nursing as a phase which might later in the history of the community shrink into one of less importance, while the nurse's rôle as herald of health grew more and more prominent. Under the new and comprehensive system of promoting national health suggested by the Consultative Council, we note with great satisfaction that the imperative necessity for bringing higher ideals of health within reach of the people is recognised.

In rather ponderous language the Report informs us that physical culture may be either constructive, or educational, or recreational, or remedial, that it aims at producing a healthy and strong body with balance and grace of movement; brain control—quick and perfected response and concentration of purpose—the joy of achievement; discipline, loyalty, comradeship. In claiming that physical culture rightly applied can achieve some if not all of these aims, the Council is supported by the experience of all who have experimented in this direction. Were sufficient energy directed to building up the physique of individuals before their health deteriorated under the exacting conditions of their lives, the huge and costly apparatus constructed to relieve them after disease has been admitted to the system could undoubtedly be largely reduced. The policy hitherto has been to let people fall freely from the crumbling banks of health into the dangerous rapids of disease and only pay attention to their rescue when they are carried off their feet, battling for their lives against the current. The need, far too long neglected, is to shore up the banks and fence the worst spots, and we welcome every sign that the Ministry of Health is alive to this grave lack in our health organisation.

The direct part which the nurse can play in building up a healthier generation possessed of a strong resistive force to disease is still very imperfectly understood. The private nurse often gets so swamped in preoccupations about disease that she acquires a deadly belief in it as an almost normal contingency waiting to engulf even the strongest without warning, without ascertainable cause. Instead of combating the popular conception of illness as a thing always in readiness to pounce just round the corner, she may be seen succumbing to this superstitution, even rapping the table to avert the omen if she is complimented on looking well. She does not in fact believe in health as a natural and Divine gift, and in losing her faith in health she has unconsciously forfeited one of her highest prerogatives as a nurse.

It is of the first importance that the women who are to carry forward the banners of health should themselves be disciplined in the doctrines they are to expound. Unless our system of training nurses gives us "healthy and strong bodies with balance and grace of movement," unless it ensures "quick and perfected response and concentration of purpose," unless it develops in the nurse "the joy of achievement, loyalty, and comradeship," it has largely failed in its purpose of training women to be pioneers of health.

Physical culture, the cult of health in contradistinction to concentration on disease, ought to hold a far more prominent part in the training-schools than it does. It is necessary in order to protect nurses in the fatigues of their training. It is necessary in order to show them one of the secrets of maintaining health under cramped or unsatisfactory conditions when their training is ended. It is necessary in order to make them centres of sound instruction wherever they go, for it is certain they will for ever be encountering those who are standing on a crumbling bank, but could recover their equilibrium if wisely taught. It will be a disastrous thing if the nursing profession drifts into two sections, one for disease and one for health. Only by those who know the secrets of a healthy life can disease be fairly met and subdued. In every branch of nursing a knowledge of physical culture, of the forces which promote vigour and repel disease is essential to the nurse.

Physical culture in the training-scheme need not be a very exacting affair. It involves the practice of co-ordinated movements under a trained instructor, but it makes no great demands on either time or money. Its further development requires grounds suited for recreational purposes, but these are lacking in few institutions. The interim report states that "the gain in improved health of the people and the diminution of illness would more than repay the cost." Substituting the word "nurses" for "people" all training-schools which introduce physical culture would find this hold good.

